# The Examination of "Leavers"

**Published:** 1913-10-25

**Authors:** 


### THE SCHOOL DOCTOR.

### The Examination of "Leavers."

Under existing conditions arrangements are generally made for the medical inspection of school children in three primary groups—*i.e.* (1) entrants, that is to say, children who are commencing their school life; (2) children attending school, usually taken from a specified age group; and, finally (3), "leavers," or children who are about to leave school. The object and aim of these three examinations are not quite the same. The first examination is a general one, designed to see in how far the child is fit for school. At it special attention is directed to defects which are likely to interfere with school life or which, in the words printed on the London County Council advice cards, "are likely to affect not only progress in school, but future prospects in life." Such defects, where remediable, are supposed to be remedied during the child's school life. At the second examination, two or three years later, the general health of the child is again considered. By this time it may have been found that the former entrant is physically or mentally defective in so far that he or she is incapable of receiving benefit in an ordinary school. In such cases a special examination will be asked for in order to find out if the child is a suitable case for admission into a "special" school. With this class of case we propose to deal in a future article. The third, or final examination, takes place when the child is about to leave school. Usually the school-leaving age is that fixed by law—namely, fourteen years, but in special circumstances boys or girls leave school earlier; those who attend secondary or central schools leave, or are at least supposed to leave, a couple of years later. As a matter of fact the average school-leaving age in the central schools is much nearer fourteen than sixteen. The school doctor, at the final examination, will therefore have before him such children as are "due to leave" within a few months, or at the end of the school year. Most of these he will have seen at least once before. Their record cards will in all cases be available for reference, and, in addition, he will be able to draw upon the knowledge of the class and head teachers for such additional information as he may require. With this record and information before him he is in a much better position to give an opinion as to the health and physical condition of the child than he is often able to do in the case of an entrant.

The "leavers'" examination is directed not so much to find out what is the child's condition with regard to suitability for school life, but what it is with reference to the work the child will have to do, and the environment he or she will be placed in when school is left behind and the workaday business of life is started. It is, therefore, necessary to find out what is likely to be the boy's or girl's career; what work he or she is inclined to take up on leaving school. Very often no definite information can be obtained on this point, and all that the medical inspector need be concerned about, then, is to find out whether or not the child is in average good health such as will fit it for any of the occupations which young people of school-leaving age may take up. The class to which the child belongs, and the nature of the work it has in mind, will have to be taken into consideration. Each case must be individually judged, but a few general points may here be noted as demanding special attention at the examination.

Obvious defects should be pointed out to the parents and every effort should be made to have such defects remedied before the child leaves school.

It is usually sufficient to draw attention to the fact that while at school the child can easily get leave to attend a hospital, private practitioner, or clinic for treatment; when work has been taken up such opportunities are infrequent and may involve considerable loss of time and money. The defect least likely to be remedied is carious teeth, and yet this is one of the defects which ought to be seen to, since it is one that may very considerably hamper the working capacity of the average boy or girl. During school-going age most carious teeth may yet be saved by judicious filling; later on dental treatment becomes much more difficult and consequently expensive. Tonsils and adenoids, if not discovered at the first examination, should be carefully examined; if operation is called for it should be pointed out to the parents that such treatment, when well carried out, considerably adds to the child's chances of obtaining employment later on. Employers, although not students of Lavater, have learned to recognise the "adenoid face" and prefer to employ boys who have a less vacant expression.

A very serious condition is a heart lesion, however adequately it may be compensated. The utility of the child's life depends entirely on the manner in which such compensation is supported and strengthened. While it is useless and foolish to alarm parents by laying stress on the miseries of "failing heart" when compensation breaks down, a word of warning is often in season to prevent the child suffering from such a lesion being seriously injured by work that is beyond its strength or cardiac capacity. Necessity is here often a very hard taskmaster, but head teachers and Care Committee members do excellent work in advising such children and securing for them employment which gives them a fair wage and at the same time brings them no danger of overstraining their heart. Clerkships, office work, and outdoor work (when not involving heavy manual work) for boys, and similarly less arduous employment for girls, are the occupations best suited for children with a compensated heart lesion. It must be borne in mind, however, that in children heart disease usually means under-development, not only of the body but of the organs as well, and some care must, therefore, be taken to prevent the child from being placed in an environment where such under-development is accentuated by bad and impure air, little food, and worry. Where an out-of-door occupation can be secured it is by far the best, even when the wages offered are less than the child can obtain elsewhere. With parents eager to make the child support itself as soon as possible the question of wages is an all-important one, but the medical officer can do a great deal by judicially illustrating the evils which are attendant upon "blind alley" and heavy manual work in such cases.

Lesions of the lungs are equally important. It is among the "leavers" that incipient pulmonary tuberculosis is most likely to occur, and the doctor should be on his guard to detect the early signs. Aberrations in the breath or percussion sounds, pallor, fatigue, and want of appetite in such children are of more grave import than in the younger children and should always induce a very careful examination of the chest, supplemented in cases where there is the slightest suspicion with bacteriological and temperature tests. Where phthisis actually exists active treatment is demanded and such a child is unfit for work. The difficulty arises in cases where a diagnosis of "pretubercular condition" is made. Such children need good food, open air, plenty of sleep, and a modicum of work sufficient to keep them active, out of mischief, and contented, without causing them worry or anxiety. To find an ideal occupation for them is often a matter of great difficulty, but here again the head teachers and Care Committees, in touch as they are with the various bodies and organisations of labour, can do a great deal to help in selecting suitable employment. The business of the school doctor is to suggest what kind of work the boy or girl is best fitted for, and to advise the parents and teachers, and the child itself, accordingly. That is indeed all he can do, for he cannot be turned into a labour agent, and his voluntary efforts in the latter capacity are more likely to bring him worry and trouble than thanks and pleasure.

Another defect that should be carefully looked for is otorrhœa. It is a condition often overlooked by the parents or by the child and regarded as of no importance; in fact, it is well known that there exists a popular superstition (not confined to the parents alone but prevalent among teachers as well) that so long as an ear discharges "freely" no harm can result, the suppuration being apparently looked upon as a necessary evacuant for all evil. Medical men are fully aware of the harm done by this superstition, and school doctors should lose no opportunity of fighting against it. Possibly the best way is to point out to the parties concerned that life assurance offices now recognise the danger of this condition, and to lay stress on the economic disadvantages resulting from such recognition. This is a much better way than to draw for the imagination a vivid picture of the possibilities of abscess of the brain as some school doctors are in the habit of doing.

Other defects to be looked for are hernia, chronic appendicitis (by no means uncommon, although exceedingly difficult to get remedied by the only radical method—operation), flat feet, and lateral curvature, defective vision (especially slight degrees of astigmatism), and chronic skin eruptions, of which acne and psoriasis are the chief.

